# Death of Dr. Hartshorne

**Published:** 1850-10

**Authors:** D. Francis Condie

**Affiliations:** Sec’y


### DEATH OF DR. HARTSHORNE.

Died at *Brandywine Springs*, near Philadelphia, on the 20th of August, Dr. Joseph Hartshorne, aged 71 years.

Dr. H. was one of the oldest practising physicians in Philadelphia, and until within a short period of his death was actively engaged in professional duties. The virtues of his character, and the high estimation in which he was held by his associates, are better expressed by the subjoined resolutions, than by any words of ours; to them, therefore, we eave the grateful duty of commemorating them.

At a session of the College of Physicians of Philadelphia, specially convened on the 26th of August, 1850, that the Fellows might have an opportunity to take such action in reference to the loss they had sustained in the demise of their late colleague, Dr. JOSEPH HARTSHORNE, as their feelings should dictate: the Vice President, Dr. Charles D. Meigs, introduced the subject of the meeting by a few remarks on the professional standing and private worth of the deceased; after which, the following Resolutions were presented by a Committee, consisting of Drs. Bell, Parrish, and Hallowell, appointed to prepare them, and unanimously adopted.

*Resolved,* That the College learn with deep emotion, the melancholy announcement of the death of its esteemed Fellow, Dr. JOSEPH HARTSHORNE.

*Resolved,* That in giving utterance to their feelings at the event, the Fellows of the College are sure to express, at the same time, those of the Profession of which the deceased was so long an eminent and esteemed member, and of the community amid which he toiled so faithfully and so ably.

*Resolved,* That the example given by the deceased, of devotion to his professional duties; of great skill joined to a frank and manly bearing in the exercise of them, be received by us as an example worthy our imitation.

*Resolved,* That a Fellow of the College be appointed to prepare a biographical notice of the deceased, to be read before the College, and inserted in its Transactions.

*Resolved,* That a Committee be appointed to communicate to the family of Dr. HARTSHORNE, the sympathy of the College in their bereavement.

To the gentlemen composing the Committee by whom the foregoing were prepared, was delegated the duty imposed by the last resolution.

It was further resolved, that the proceedings of the meeting be published in the Medical Journals of this city.

D. FRANCIS CONDIE, Sec'y.

